# Dynamic assessment and zoning strategies for ecosystem health in Poyang lake urban agglomeration

**DOI:** 10.1038/s41598-025-05776-6

**Published:** 2025-07-01

**Authors:** Zhiyao Zou, Songkai Luo, Ping Duan

**Affiliations:** 1 College of Land Resources and Environment, Jiangxi Agricultural University, Nanchang, 330045 China; 2 Key Laboratory of Poyang Lake Watershed Agricultural Resources and Ecology, Ministry of Agriculture and Rural Affairs, Jiangxi Agricultural University, Nanchang, 330045 China

**Keywords:** Ecosystem health, Spatiotemporal characteristic, Driving factor, Zoning control, Geodetector, Poyang lake urban agglomeration, Environmental sciences, Ecosystem services, Urban ecology

## Abstract

Assessing and appropriately managing the ecosystem health of urban agglomerations are significant for safeguarding regional ecological security and sustainable development. From a spatiotemporal dynamic perspective, with the Poyang Lake urban agglomeration as a case, this study established a ‘Vigour–Organisation–Resilience–Service’ framework to assess ecosystem health, analysed spatiotemporal characteristics, identified key driving factors and their interactions contributing to spatial differentiation and ultimately adopted a ‘level–type’ zoning control strategy. The results show that: (1) From 2005 to 2022, the natural ecosystem health index declined in 14.70% of the region, with a central concentration of low values in the Poyang Lake area. The ecosystem service index decreased in 11.10% of the region but remained high in the Poyang Lake area. Varying degrees of positive spatial spillover effects were observed for both indices. (2) The average regional comprehensive ecosystem health index was 0.2914, with an overall downward trend. In the urban agglomeration, the comprehensive ecosystem health index remained stable in 78.18% of the total area, indicating a notable ‘polarisation’ in health levels among different areas. (3) Socioeconomic factors significantly influence ecosystem health in the urban agglomeration. (4) The urban agglomeration displayed distinct spatial patterns in terms of the types of changes in ecosystem health. Considering both current status and trends, zoning control and differentiated management strategies were implemented for the urban agglomeration. The approaches of differentiated management and zoning control can promote the coordinated development of the Poyang Lake urban agglomeration, facilitating a dynamic balance between urbanisation and ecological protection.

**Supplementary Information:**

The online version contains supplementary material available at 10.1038/s41598-025-05776-6.

## Introduction

Ecosystem health refers to the capacity of an ecosystem to stably and sustainably provide goods and services necessary for human society, emphasising its resilience to external impacts on its structure and functions^1,2^. In this respect, the zoning management of ecological security is crucial for safeguarding ecosystem health, particularly through the enhanced assessments of changes in ecological functions and the monitoring of ecological security risks in key ecological security zones, thereby preserving ecological barriers. However, with population growth, urbanisation and rapid socioeconomic development, the state of ecosystem health is faced with increasing challenges^3^. Therefore, accurately assessing regional ecosystem health, comprehending its inherent logic and implementing zoning management have become key issues for ensuring ecological security and enhancing regional ecological quality.

Currently, research on ecosystem health in urban agglomerations is at a nascent and exploratory stage both domestically and internationally, focusing on four key topics: (1) regional research on the assessment and spatial characteristics of ecosystem health^4^; (2) discussions about ecosystem health assessment indicator systems^5^; (3) formulation of zoning and management strategies based on ecosystem health^6^ and (4) investigations into the mechanisms influencing ecosystem health^7,8^. From the perspective of urban ecology, urban agglomerations represent a complex social, economic and natural ecosystem. They are also the most dynamic and promising frontier for regional and socioeconomic development, where cities, residential areas and supporting systems form an intricate structure simulating the functions of natural ecosystems^9^. At this stage, research on ecosystem health mostly focuses on natural systems, such as rivers^10,11^lakes^12,13^grasslands^14,15^forests^16,17^ and wetlands^18,19^. Thus, the ecosystem health of complex urban agglomerations in the social system must be further explored.

Analysing the services and health of ecosystems in urban agglomerations can provide new technical frameworks and theoretical guidance for optimising their territorial ecological restoration. Moreover, existing ecosystem health evaluation systems incorporate multiple perspectives, including the Pressure–State–Response model^9^the Driver–Pressure–State–Impact–Response model^20^ and the Vigour–Organisation–Resilience (VOR) ecosystem assessment model^21^. While frameworks like the VOR model provide insights into the ecosystem structure, they inadequately account for the interactions between ecosystem services and human well-being, and often neglect the complexity of socio-natural coupled systems in urban agglomerations. Therefore, A critical direction for future ecosystem health assessments is the further elucidation of the logical relationships between ‘vigour, organisation, resilience and ecosystem services’^22^consideration of the support and importance of ecosystems for the development of human society and exploration of the connections and integration between ecosystem services and VOR^23^. Furthermore, ecosystem health assessments partially mirror the impacts of land-use changes and urbanisation on ecosystem health, which has great significance for attaining regional sustainability and fostering synergy between ecosystem health and the development of urban agglomerations.

Existing research suggests that disparities in ecosystem health among different regions are shaped by diverse factors, most of which stem from social systems, spatially varied natural systems and their interplay^24^. Scholars have predominantly adopted approaches such as principal component analysis^25,26^grey relational analysis^27,28^and Geodetector^8,29,30^ to delve into the impacts of these factors. In recent years, Geodetector has enabled the quantitative analysis of driving factors behind geographical phenomena, assisting in the identification and interpretation of spatial differences attributed to geographical effects. However, although it has been widely applied in ecological and environmental research, it remains underutilised in ecosystem health research and analysis^31^. In this field, rapid urbanization in urban agglomerations has intensified ecological pressures through population growth and economic expansion. However, few studies have explored the nonlinear effects of socioeconomic factors and their interactions with ecological indicators^31^.

Further, the application of the ‘level–type’ integrated zoning and management strategy is a key focus among the results of investigations of driving mechanisms and ecosystem health assessments. The level–type approach is more commonly employed in the fields of watershed ecology^32^farmland patterns^33^ and ecological risk assessments^34,35^among others, with limited application in the management of urban agglomerations that combines ‘current health’ with ‘types of changes’. The implementation of this zoning strategy could diversify approaches to the management of ecosystem health in urban agglomerations and facilitate regional ecological conservation and high-quality development^6^.

In summary, previous research on ecosystem health mostly focused on natural ecosystems and watersheds, but there are few studies on socio-natural complex systems. Although existing ecosystem health assessment systems incorporate multiple dimensions, they fail to sufficiently consider the significance of ecosystem services in social development. Moreover, most studies delved into methods for exploring factors, whereas research on the driving factors for ecosystem health and their interactions remains scarce. In urban agglomerations, the integration of level–type zoning with the formulation of management strategies must be further deepened.

Hence, this study evaluates the ecological health status of the Poyang Lake urban agglomeration based on the principles of harmonious coexistence between humans and nature, systematic governance, and global ecological security, combined with spatial heterogeneity analysis and geodetector modeling.

With multisource data and the Poyang Lake urban agglomeration as a study area^36^this study assesses the ecosystem health of the region and conducts a quantitative analysis and zoning based on the “Vigour–Organisation–Resilience–Service” (VORS) framework, developing a ‘level–type’ zoning strategy tailored to the unique ecological-security challenges of the Poyang Lake urban agglomeration. This work identifies key driving factors impacting ecosystem health and their interactive strength using the Geodetector technology to provide a reference for environmental protection and sustainable socioeconomic development.

## Materials and methods

### Overview of the study area

**Fig. 1:**
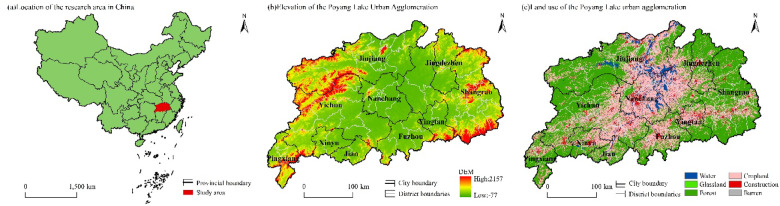
Location of the research area in China(**a**), Elevation of the Poyang Lake Urban Agglomeration(**b**) and Land use of the Poyang Lake urban agglomeration(**c**). The process of creating Figure.1 was as follows: First, (**a**), (**b**) and (**c**) were drawn in ArcGIS 10.8.1 and exported as three separate images. These images were then arranged and compiled in Adobe Photoshop 2023 to produce Figure.1.

The Poyang Lake urban agglomeration is located at 28°10′–29°51′N, 115°23′–117°45′E in the north of Jiangxi Province on the southern bank of the middle and lower reaches of the Yangtze River. This area, characterised by a subtropical monsoon climate, is a key lake and transition zone between water and land in the Yangtze River Basin (Fig. 1). This paper focuses on the Poyang Lake urban agglomeration, with Nanchang as the provincial capital, which encompasses the administrative region of 10 prefecture-level cities, such as Nanchang, Jingdezhen, Shangrao, Yingtan, Yichun, Xinyu and Pingxiang and parts of Fuzhou and Ji’an and 64 counties (cities), covering a total area of 92,300 km^2^. In 2021, the permanent population in the Poyang Lake urban agglomeration was ~ 30.6679 million, accounting for 67.89% of the total population in Jiangxi Province. Its regional GDP amounted to 1,945.99 billion yuan, constituting 65.70% of the total GDP of Jiangxi Province, with an overall urbanisation rate of 61.46%.

The terrain of the region predominantly comprises mountains and plains, with the particularly low and flat Poyang Lake Plain at its centre encircled by mountains. Land use is dominated by forestlands and croplands. Forests are concentrated in the western Jiuling Mountains, the eastern Huaiyu Mountains and the southeastern Wuyi Mountains. Croplands are mainly distributed in the north and central areas of the Poyang Lake Plain. Water areas include the Ganjiang River, the Fu River, the Xiu River, the Xin River, the Rao River and Poyang Lake. Construction land is chiefly concentrated in economically vibrant and densely populated urban areas, such as Nanchang, Jiujiang, Yichun and Fuzhou. As urbanisation in the Poyang Lake urban agglomeration accelerates and territorial space is developed excessively, environmental problems such as wetland shrinkage and biodiversity loss​​, hydrological disruption, and land-use conflicts have become increasingly prominent in this region.

### Data sources

This study relied on multisource data from the years 2005, 2010, 2015, 2020 and 2022, encompassing data from ecosystem health assessments and factor detection by Geodetector. The data used in this study include land use data, meteorological data, topographic data, vegetation data, socio-economic data and other multi-source data, as shown in Table 1 (For more detailed data, please refer to Supplementary Material Table 1). The changes in land use and land cover are sourced from the Chinese Land Cover Dataset (CLCD)^37^. Slope calculated using DEM, which is obtained from the European Space Agency. The net primary productivity (NPP) and normalized difference vegetation index (NDVI) datasets are derived from the MOD17A2H^38^ and MOD13A3^39^ products available through Google Earth Engine, respectively. Precipitation dataset is computed based on monthly data^40^and population density is represented as a grid dataset at a one-kilometer resolution, organized according to administrative divisions. Per capital farmland area calculated using LUCC and population density. Land urbanization degree is calculated using LUCC and population density. Other socio-economic data were obtained from relevant city, county, and district statistical yearbooks and supplemented by consultations with local government authorities.

To better capture the spatial variability of ecosystem health in the Poyang Lake urban agglomeration, the study area was divided on a grid scale. Following ecological grids built by other scholars in similar studies^4,41^and considering the actual area of the Poyang Lake urban agglomeration, this study adopted a 3 × 3 km grid to divide the study area into 10,698 assessment units, and the data were unified to each study unit using spatial analysis methods such as partition statistics and area tabulation.

**Table 1:** Data source and precision.

Type	Data source
Land use/land cover data	https://zenodo.org/record/8176941
Normalized Difference Vegetation Index(NDVI)	https://e4ft101.cr.usgs.gov/
Net primary productivity of vegetation(NPP)	
Annual average precipitation	https://data.tpdc.ac.cn/
Annual average temperature	
Average nighttime light index	http://www.resdc.cn/
Digital elevation model	https://panda.copernicus.eu/panda
Population density	https://landscan.ornl.gov/
Administrative district	https://www.webmap.cn/main.do?method=index
Other socio-economic data	Corresponding city, county, and district statistical yearbooks and consultation with local governments

### Research methods

#### Building of an ecosystem health assessment model

The VORS ecosystem health assessment model adopted herein accounted for the various services and products that ecosystems provide for human development while focusing on the cascading effects of vigour–organisation–resilience within these systems. The inclusion of ecosystem services enhances the framework’s ability to reflect human well-being and adapt dynamically to rapid urbanisation and land use changes, as shown in the following equation:

1 $$\:HI=\sqrt{PH\times\:CESI},$$

where *HI* denotes the ecosystem health level, *PH* represents the natural ecosystem health index, and *CESI* stands for the comprehensive ecosystem service index.

In terms of limitations, subjectivity in indicator selection and weighting was addressed by carefully selecting indicators relevant to the study area. High-quality data sources and implemented validation procedures were implemented, including cross-referencing with local records and field surveys.

(1) Natural Ecosystem Health Index.

Ecosystem organisation, vigour and resilience^5,21,42^ are all key indicators in ecosystem health assessments, each assigned equal weight based on relevant literature^23^as shown in the following equation:

2 $$\:PH=\sqrt[3]{EV\times\:EO\times\:ER},$$

where *PH* signifies the natural ecosystem health index and *EV*, *EO* and *ER* represent ecosystem vigour, organisation and resilience, respectively, which are also normalised.

*EV* refers to the vitality, metabolism or primary productivity of an ecosystem^43^often represented by the NDVI^44^ and is crucial for assessing ecosystem health and sustainability.

In ecosystem analysis, *EO* serves as a key indicator for assessing the structural stability and complexity of a regional ecosystem, encompassing landscape heterogeneity (*LH*), landscape connectivity (*LC*) and important patch connectivity (*IPC*)^9^. A quantitative analysis is conducted based on previous research^45^;^46^ with the following equation:

3 $$\begin{array}{l}\:{\rm{EO = 0}}.{\rm{35}}\:{\rm{LH + 0}}.{\rm{35}}\:{\rm{LC + 0}}.{\rm{30}}\:{\rm{IPC = }}({\rm{0}}.{\rm{25}}\:{\rm{SHDI + 0}}.{\rm{10}}\:{\rm{AWMPFD)}}\\\quad \quad\,\,\,{\rm{ + }}({\rm{0}}.{\rm{25}}\:{\rm{F}}{{\rm{I}}_{\rm{1}}}{\rm{ + 0}}.{\rm{10}}\:{\rm{CONT) + }}\left( {{\rm{0}}.{\rm{10}}\:{\rm{F}}{{\rm{I}}_{\rm{2}}}{\rm{ + 0}}.{\rm{05}}\:{\rm{COHESIO}}{{\rm{N}}_{\rm{1}}}} \right){\rm{ + }}\left( {{\rm{0}}.{\rm{10}}\:{\rm{F}}{{\rm{I}}_{\rm{3}}}{\rm{ + 0}}.{\rm{05}}\:{\rm{COHESIO}}{{\rm{N}}_{\rm{2}}}} \right),\end{array}$$

where Shannon’s diversity index (*SHDI*) and area-weighted mean patch fractal dimension (*AWMPFD*) are used for assessing the diversity and complexity of an ecosystem; the overall landscape fragmentation index (*FI*_1_) and the landscape contagion index (*CONT*) represent the degree of landscape fragmentation and the tendency of contagion; the water fragmentation index and the forestland fragmentation index (*FI*_2_ and *FI*_3_, respectively) are used to measure the degree of water and forestland fragmentation, respectively; and the water patch cohesion index and the forestland patch cohesion index (*COHESION*_1_ and *COHESION*_2_) are used to evaluate the cohesion of water and forestland patches.

*ER* refers to the ability of an ecosystem to maintain its original structure and functions under stress, mainly manifested as its resilience to external impacts and embodied in the two aspects of resistance and resilience. The coefficient weights are determined based on existing literature^47–49^ using the following equation:

4 $$\:ER=0.4\times\:\sum\:_{i=1}^{n}{A}_{i}\times\:{C}_{\text{r}\text{e}\text{s}\text{i}\text{s}\text{t}\text{a}\text{n},\text{i}}+0.6\times\:\sum\:_{i=1}^{n}{A}_{i}\times\:{C}_{resilien,i},$$

where *A*_*i*_ represents the area proportion of type *i* of land use in the region, ranging from 1 to 6, corresponding to farmland, forestland, grassland, waters, construction land and bare land, respectively; and *C*_resistan,*i*_ and *C*_resilien,*i*_ represent the resistance and resilience coefficients of type *i* of land use, respectively.

(2) Ecosystem Service Index.

The alignment between the coordination of ecosystem service supply and demand and human well-being has become a key field and direction of research on ecosystem services^50^. To delineate the magnitude of ecosystem service capabilities in the Poyang Lake urban agglomeration, this study calculated the area-specific ecosystem service value (*AESV*)^51^ and normalised ecosystem service value across grid cells^41^ to derive the CESI. The equations are as follows:

5 $$\:ESV=\sum\:_{i=1}^{n}({U}_{i}\times\:{\text{V}\text{C}}_{i})$$

6 $$\:AESV=\sum\:_{i=1}^{n}({U}_{i}\times\:{\text{V}\text{C}}_{i})/\sum\:_{i=1}^{n}{U}_{i},$$

7 $$\:CESI=\frac{AESV-{AESV}_{min}}{{AESV}_{max}-{AESV}_{min}},$$

where *ESV* denotes the ecosystem service value, *U*_*i*_ represents the area of the type *i* ecosystem, and *VC*_*i*_ signifies the service value of this type. Meanwhile, *AESV* is an average indicator of the area-specific ecosystem service value in the assessed region. *CESI* represents the comprehensive ecosystem service index, with *AESV*_max_ and *AESV*_min_ denoting the highest and lowest area-specific ecosystem service values, respectively.

(3) Ecosystem Health Level.

On the basis of the quantified results of ecosystem service functions in the Poyang Lake urban agglomeration, following established practices^23^this paper adopted the method of natural breaks to categorise the results in the years 2005, 2010, 2015, 2020 and 2022 into five levels: low, relatively low, moderate, good and excellent. In the context of ecosystem health classification, the method of natural breaks is intuitive, comparative and goal-oriented, facilitating a clearer depiction of ecosystem service function states across various years or regions and enabling quick identification of trends and differences in these functions.

#### Spatial differentiation and driving factor analysis

To explore the temporal differentiation characteristics of the Poyang Lake urban agglomeration, this study utilised the global Moran’s I to represent the spatial autocorrelation of ecosystem health and hotspot analysis^30^ to grasp its spatial distribution and pattern of changes. The Geodetector model was applied to the recognition of the key driving factors influencing the spatial heterogeneity of ecosystems. The equations are detailed in Table 2.

**Table 2:** Analytical methods for driving factors and Spatial heterogeneity.

Specific Research Methods	Definition and Role	References
Global Moran’s I	Global Moran’s I is a statistical measure used to assess the degree of spatial autocorrelation across an entire study area. It determines whether observed spatial patterns (e.g., clustering or dispersion) deviate significantly from randomness.	^52,53^
Hotspot analysis	This method identifies localized spatial clusters (hotspots or coldspots) where values are statistically higher or lower than expected under spatial randomness.	^54,55^
Geodetector	Geodetector evaluates the explanatory power of driving factors and their interactions on spatial phenomena, leveraging spatial stratified heterogeneity principles.	^7,8,56^

Interaction detection is to identify the interaction between different risk factors Xs, that is, to evaluate whether the combined effect of factors X1 and X2 will increase or decrease the explanatory power of the dependent variable Y, or whether these factors have independent effects on Y. The types of interactions are shown in Table 3.

**Table 3:** Types of interaction between Geodetector.

Interaction type	Definition	Q-value relationship
Dual factor enhancement	When two factors act together, their explanatory power for the dependent variable is stronger than when they act separately.	$\:\text{q}({\text{X}}_{1}\cap\:{\text{X}}_{2})>\text{m}\text{a}\text{x}\left(\text{q}\right({\text{X}}_{1}),\text{q}({\text{X}}_{2}\left)\right)$
Nonlinear enhancement	When two factors act together, their explanatory power for the dependent variable is stronger than when they act alone, and the sum of their explanatory power alone is less than the explanatory power of their combined effect.	$\:\text{q}({\text{X}}_{1}\cap\:{\text{X}}_{2})>\text{q}\left({\text{X}}_{1}\right)+\text{q}\left({\text{X}}_{2}\right)$
Nonlinear attenuation	When two factors act together, their explanatory power for the dependent variable is weaker than when they act separately.	$\:\text{q}({\text{X}}_{1}\cap\:{\text{X}}_{2})<\text{m}\text{i}\text{n}\left(\text{q}\right({\text{X}}_{1}),\text{q}({\text{X}}_{2}\left)\right)$
Single factor nonlinear attenuation	One factor has a stronger explanatory power for the dependent variable, while the other factor has a weaker explanatory power for the dependent variable. When the two factors work together, their explanatory power for the dependent variable is between their individual effects.	$\:\text{m}\text{i}\text{n}\left(\text{q}\right({\text{X}}_{1}{),\text{q}(\text{X}}_{2}\left)\right)<\text{q}({\text{X}}_{1}\cap\:{\text{X}}_{2})<\text{m}\text{a}\text{x}\left(\text{q}\right({\text{X}}_{1}{),\text{q}(\text{X}}_{2}\left)\right)$
Independence	The influence of two factors on the dependent variable is independent of each other, and when they act together, their explanatory power on the dependent variable is equal to the sum of their explanatory power when they act alone.	$\:\text{q}({\text{X}}_{1}\cap\:{\text{X}}_{2})=\text{q}\left({\text{X}}_{1}\right)+\text{q}\left({\text{X}}_{2}\right)$

#### Classification of ecosystem health levels and control zones

(1) Classification of Ecosystem Health Levels.

The comprehensive ecosystem health is classified into five levels: low, relatively low, moderate, fairly good and excellent. To better understand the trends of these health levels over time, this study compared comprehensive health levels periodically, calculating the difference between consecutive periods to derive four sets of outcomes reflecting their changes^6^. On this basis, there were five types of dynamic changes in health levels: continuous decline (Type I), fluctuating decline (Type II), stability (Type III), fluctuating increase (Type IV) and continuous increase (Type V) (Table 4).

8 $$\:\varDelta\:{L}_{t}={L}_{t+1}-{L}_{t},$$

9 $$\:{S}_{L}=\sum\:_{t=1}^{n}\varDelta\:{L}_{t},$$

where *L* denotes the comprehensive health level, Δ*L* represents the changes in the comprehensive health level, *t* indicates the study period, and *S*_*L*_ is the cumulative changes in the comprehensive health level.

**Table 4:** Types of dynamic changes in health levels.

Types of dynamic changes in health levels	Range	Characteristics
Continuous decline type	$\:\varDelta\:{L}_{t}\le\:0\wedge\:{S}_{L}<0$	The trend of continuous decline in health level
Fluctuation decline type	$\:\exists\:\varDelta\:{L}_{t}>0\wedge\:\exists\:\varDelta\:{L}_{t}<0\wedge\:{S}_{L}<0$	The health level fluctuates but overall declines
Stable type	$\:\varDelta\:{L}_{t}=0\wedge\:{S}_{L}=0$	Relatively stable health level
Fluctuation rise type	$\:\exists\:\varDelta\:{L}_{t}>0\wedge\:\exists\:\varDelta\:{L}_{t}<0\wedge\:{S}_{L}>0$	The health level fluctuates but overall increases
Continuous rise type	$\:\varDelta\:{L}_{t}\ge\:0\wedge\:{S}_{L}>0$	Continuous upward trend in health level

(2) Ecosystem Health Control Zones.

To directly reflect the comprehensive health level of the Poyang Lake urban agglomeration, this study took the Jiangxi Province Ecology and Environment Zoning Control Guidelines (2023) issued by the Department of Ecology and Environment of Jiangxi Province in 2024 for reference. Considering that counties, as fundamental administrative units, have certain authority and capabilities of resource allocation, this study adopted counties as the unit for controlling the comprehensive health level. The comprehensive health level was categorised into five classes: I_a_, II_a_, III_a_, IV_a_ and V_a_, whereas level changes were classified into five types: I_b_, II_b_, III_b_, IV_b_ and V_b_. By integrating the current health levels with the types of their changes, this study identified 23 level–type combinations^6^. These combinations enabled the implementation of more targeted zoning control strategies for emergency control zones, strict control zones, dynamic regulation zones, preventive management zones and sensitive monitoring zones (Table 5).

**Table 5:** Comprehensive health level control zones.

Control Zones	Level–Type Combination	Characteristics
Emergency control zone	IV _a_—V _b_, V _a_—V _b_, V _a_—IV _b_, IV _a_—IV _b_	Areas at a low current health level and of a level type in continuous decline
Strict control zone	V _a_—II_b_, V _a_—III_b_, IV _a_—I _b_, IV _a_—II_b_, IV_a_—III_b_	Areas at a low current health level but of a level type tending to be stable
Dynamic regulation zone	III_a_—I _b_, III_a_—II_b_, III_a_—III_b_, III_a_—IV_b_, III_a_—V _b_	Areas at a stable health level and of a relatively stable level type
Preventive management zone	I _a_—II _b_, I _a_—IIII _b_, I _a_—IV _b_, III_a_—II_b_, III_a_—III_b_, II _a_—IV _b_	Areas at a high current health level and of a level type in decline/remaining stable
Sensitive monitoring zone	I _a_—I _b_, II _a_—I _b_, II_a_—V _b_	Areas at a high current health level but of a level type changing drastically

## Results and analysis

### Spatiotemporal evolution of ecosystem health in the Poyang lake urban agglomeration

#### Spatiotemporal variations in the natural ecosystem health index

**Fig. 2:**
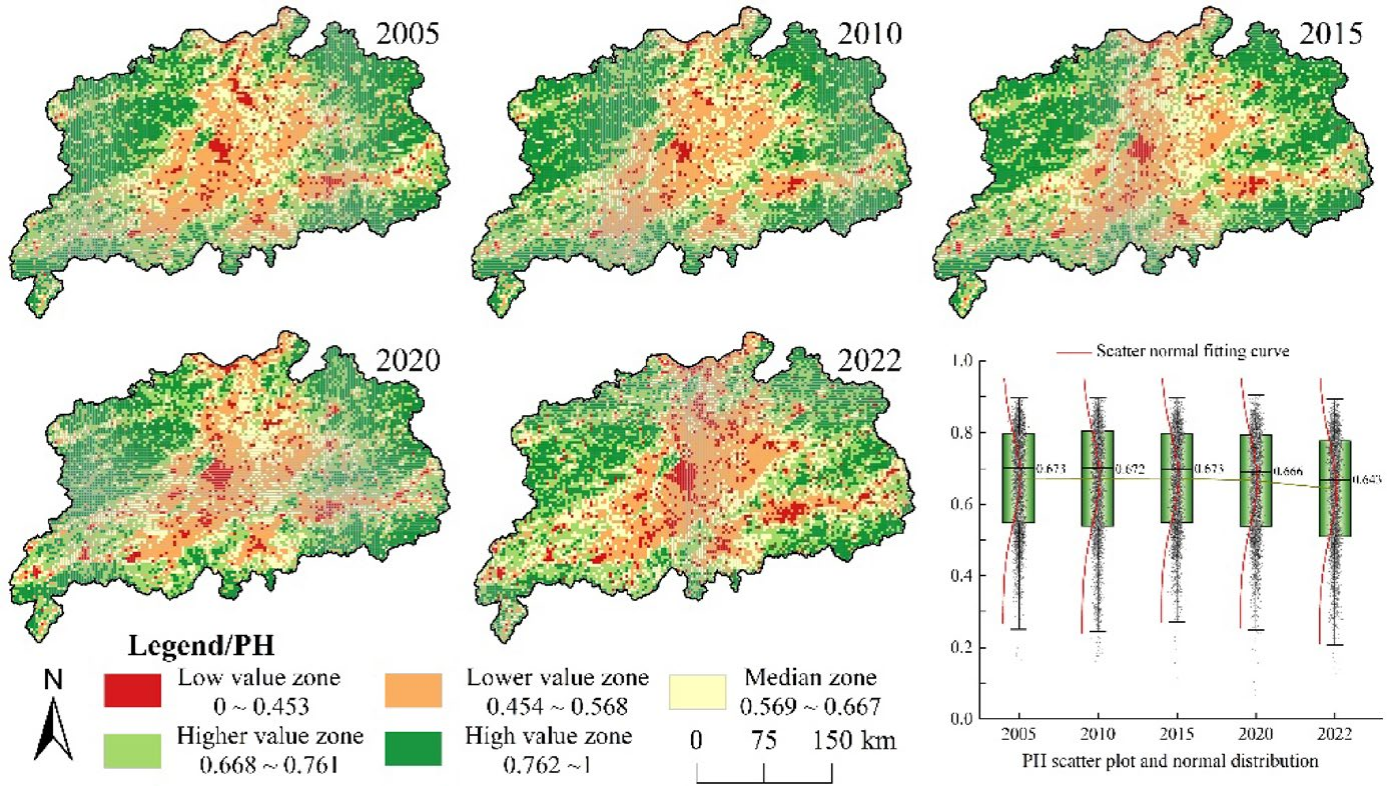
Spatial temporal differentiation of the natural ecosystem health index in the Poyang Lake urban cluster. The process of creating Fig. 2 is as follows: First, calculate the natural ecosystem health index of the study area in ArcGIS 10.8 for the years 2005, 2010, 2015, 2020, and 2022, and export the results as five separate images. Then obtain the PH scatter plot and normal distribution in Origin 2023, and export the results as separate images. Finally, arrange and compile in Adobe Photoshop 2023 to generate Fig. 2.

The natural ecosystem health index (PH) of the Poyang Lake urban agglomeration was categorized into five intervals using the natural breaks method: high (0.762–1.000), relatively high (0.668–0.761), moderate (0.569–0.667), relatively low (0.454–0.568), and low (0–0.453) (Fig. 2). Spatially, PH exhibited higher values in mountainous regions and lower values in central plains, with clustered distributions of extreme values. In 2005, high PH areas were primarily distributed in the northeastern Huaiyu Mountains and northwestern Mufu Mountains, characterised by dense vegetation cover, robust soil and water conservation capabilities, and high biodiversity. Conversely, low PH values were concentrated in economically developed urban centers and their peripheries in the central and southern regions.Temporally, the mean PH value declined from 0.673 in 2005 to 0.643 in 2022. Between 2010 and 2015, moderate-value zones in the Poyang Lake area decreased notably, while relatively low and low-value areas expanded. By 2022, significant expansion of low and relatively low-value areas was observed in the central urban agglomeration. Areas exhibiting PH declines accounted for 14.70% of the total region from 2005 to 2022, predominantly concentrated in central Nanchang, southeastern Yichun, and northern Yingtan. This degradation was associated with urban expansion and agricultural restructuring in densely populated plains, which encroached upon water bodies and forestland. These changes in land use were closely associated with the diminishing ecosystem vigour, organisation and resilience in these areas.

**Fig. 3:**
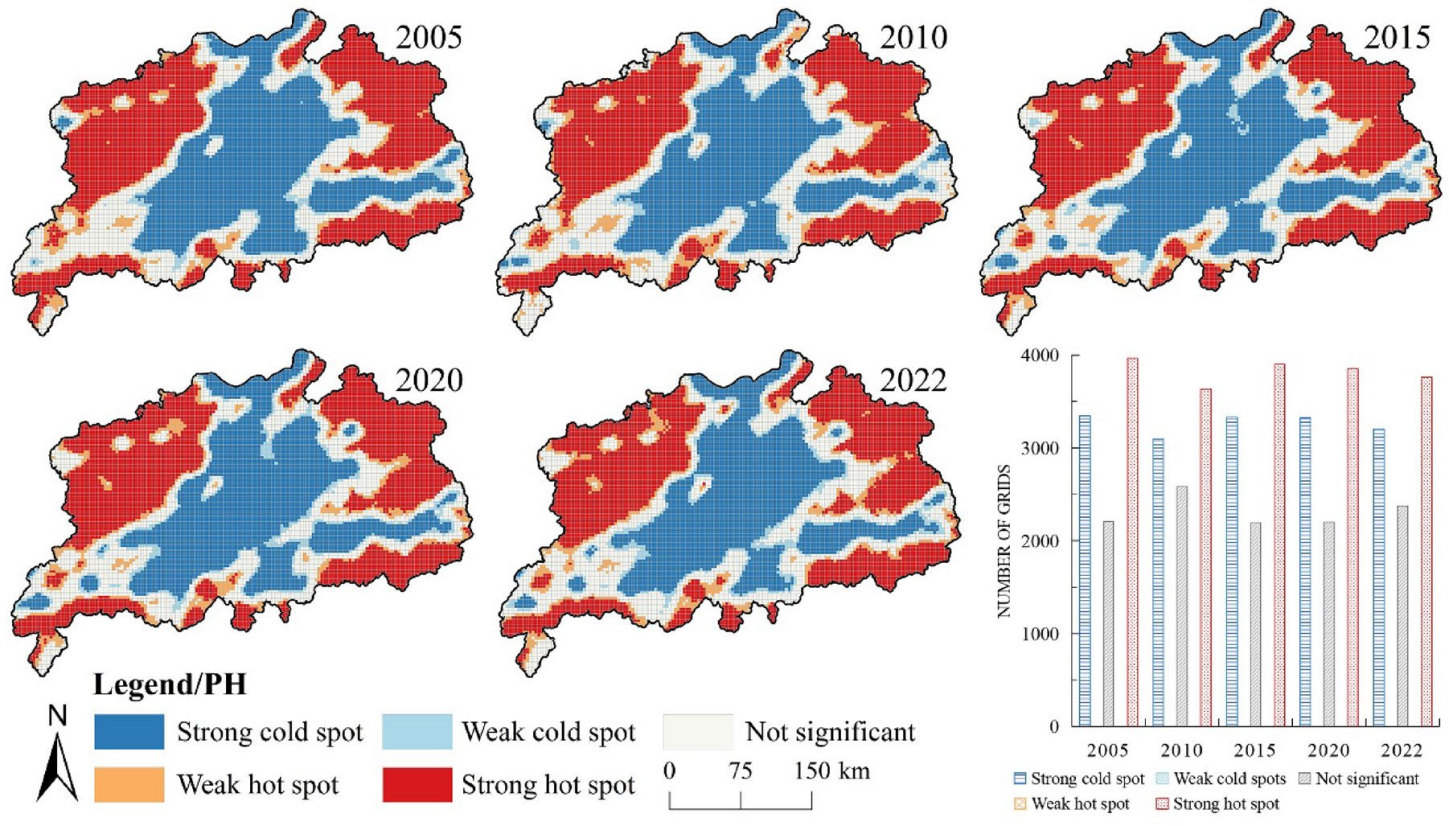
Cold and hot spot spatial distribution of the natural ecosystem health index in the Poyang Lake urban cluster. The process of creating Fig. 3 is as follows: First, calculate the cold and hot spot spatial distribution of the natural ecosystem health index for the study area in ArcGIS 10.8 for the five years 2005, 2010, 2015, 2020, and 2022, and export the results as five separate images. Then obtain the bar chart of number of grids in Microsoft Office Excel 2016 and export the result as a separate image. Finally, arrange and compile in Adobe Photoshop 2023 to generate Fig. 3.

To analyse the spatial agglomeration characteristics of ecosystem health in the Poyang Lake urban agglomeration in 2005, 2010, 2015, 2020 and 2022, global spatial autocorrelation analysis revealed significant positive spatial clustering (*p* < 0.001)(Table 6).The global Moran’s I values in these five years were 0.706, 0.593, 0.712, 0.719 and 0.663, respectively, with the highest autocorrelation in 2020 and the lowest in 2010. The map of ecosystem health hot and cold spots (Fig. 3) showed that the spatial pattern of the Poyang Lake urban agglomeration remained generally stable, displaying a distribution pattern with cold spots in the centre and hot spots at both sides. Cold spots and minor cold spots were primarily distributed at the northern and southern ends and connected areas in the centre, whereas hot spots and minor hot spots were mainly located in the northeastern and northwestern areas, gradually expanding over time. The above results may be attributed to the rapid urban development and high levels of urbanisation in the central Poyang Lake Plain area, where the natural ecosystem health level was lower. However, there were higher natural health levels in the peripheral areas with slower urban expansion and less ecological damage.

**Table 6:** Global autocorrelation test of the natural health index of the ecosystem in the Poyang lake urban agglomeration.

PH	2005	2010	2015	2020	2022
Moran’s I	0.706	0.593	0.712	0.719	0.663
z scores	101.478	85.274	102.298	103.267	95.192
P value	<0.001	<0.001	<0.001	<0.001	<0.001

#### Spatiotemporal variations of the ecosystem service index

**Fig. 4:**
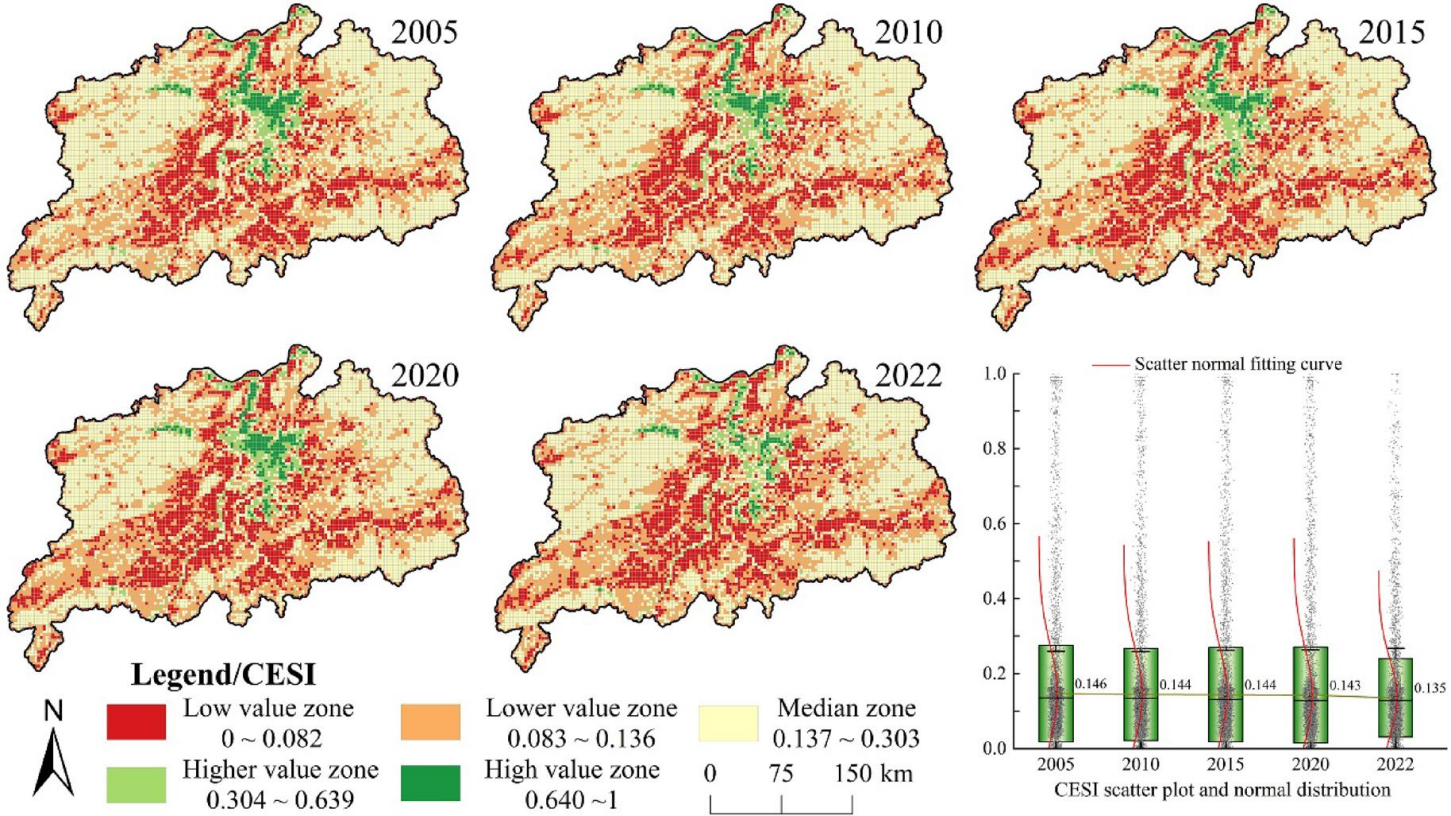
Spatial differentiation of ecosystem service index in the Poyang Lake urban agglomeration. The process of creating Fig. 4 is as follows: First, calculate the ecosystem service index of the study area in ArcGIS 10.8 for the years 2005, 2010, 2015, 2020, and 2022, and export the results as five separate images. Then obtain the CESI scatter plot and normal distribution in Origin 2023, and export the results as separate images. Finally, arrange and compile in Adobe Photoshop 2023 to generate Fig. 4.

On the basis of the analysis of the above natural ecosystem health levels, a quantitative analysis of ecosystem service functions was conducted within the study area (Fig. 4). Using the natural breaks method, the ecosystem service index was divided into five levels: the range of high values (0.640–1.000), relatively high values (0.304–0.639), moderate values (0.569–0.667), relatively low values (0.454–0.568) and low values (0–0.453). In terms of spatial distribution, the highest values of the ecosystem service index in the Poyang Lake urban agglomeration were concentrated around the lake, characterised by extensive waters and relatively flat terrain, with the value of aquatic ecosystem services occupying a significant position. Around Poyang Lake, areas with low values extended outward in a radial pattern. Examined along with the type of land use, areas with lower values of the ecosystem service index corresponded with widespread farmland in the urban agglomeration. From a temporal perspective, the mean ecosystem service index for the Poyang Lake urban agglomeration showed a clear decline from 2005 to 2022, with values of 0.146, 0.144, 0.144, 0.143 and 0.135, respectively. Between 2005 and 2022, 11.10% of the urban agglomeration experienced a decrease in the level of the ecosystem service index, whereas only 3.03% of its area showed an increase. This suggested that the overall ecosystem of the Poyang Lake urban agglomeration faced stronger external impacts and that its own resilience and self-repair capability weakened.

**Fig. 5:**
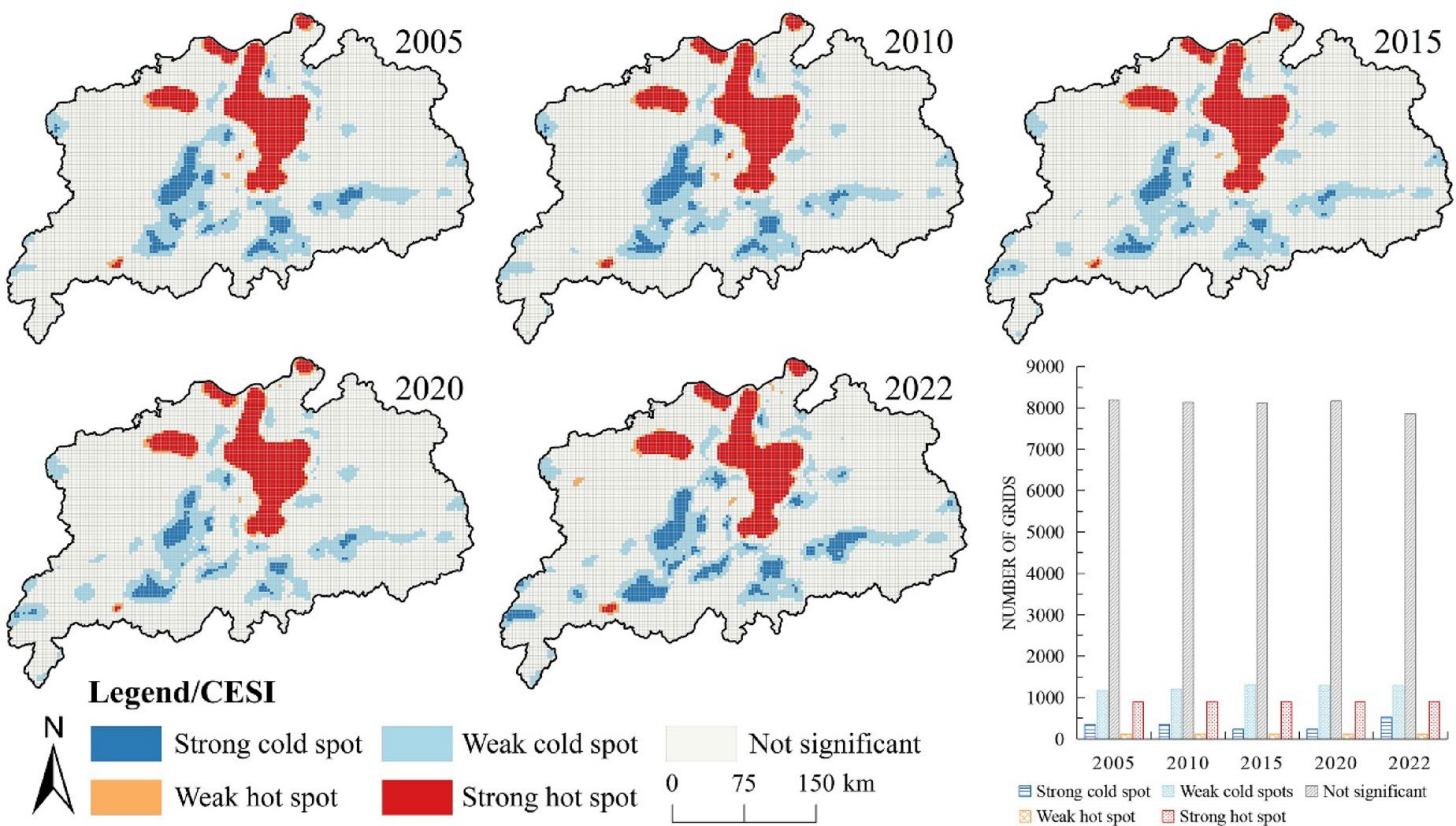
Ecosystem service index cold and hot spot spatial distribution in the Poyang Lake urban agglomeration. The process of creating Fig. 5 is as follows: First, calculate the cold and hot spot spatial distribution of the ecosystem service index for the study area in ArcGIS 10.8 for the five years 2005, 2010, 2015, 2020, and 2022, and export the results as five separate images. Then obtain the bar chart of number of grids in Microsoft Office Excel 2016 and export the result as a separate image. Finally, arrange and compile in Adobe Photoshop 2023 to generate Fig. 5.

A spatial autocorrelation analysis of the ecosystem service index was performed (Table 7), which obtained the Moran’s I values of 0.743, 0.715, 0.719, 0.734 and 0.619 for the years 2005, 2010, 2015, 2020 and 2022, respectively. The results indicated positive spatial spillover effects among various ecosystem services. Moreover, a local hotspot analysis of the ecosystem service index within the urban agglomeration was conducted (Fig. 5). The results revealed that regions such as southeastern Yichun and northwestern Fuzhou were primarily characterised by the concentration of cold and slight cold spots, which were often situated on the periphery of economically active regions. Meanwhile, regions represented by Poyang Lake and its vicinity all showed obviously concentrated hot and minor hot spots of the ecosystem service index. These may result from the generally lower values of the ecosystem health index in economically developed areas. Since 2000, the Poyang Lake region in central China has achieved rapid economic growth, along with a significant expansion of construction land and higher efficiency of land use, which led to notable environmental degradation and a decrease in the supply of ecosystem services. However, in mountainous, riverine and lacustrine habitats with less human interference, ecosystem services have been effectively conserved, resulting in distinct differences in the agglomeration of hot and cold spots of ecosystem health between these two regions.

**Table 7:** Global autocorrelation test of ecosystem service index in the Poyang lake urban agglomeration.

CESI	2005	2010	2015	2020	2022
Moran’s I	0.743	0.715	0.719	0.734	0.619
z scores	106.846	102.873	103.345	105.539	89.084
P value	<0.001	<0.001	<0.001	<0.001	<0.001

#### Ecosystem health assessment

**Fig. 6:**
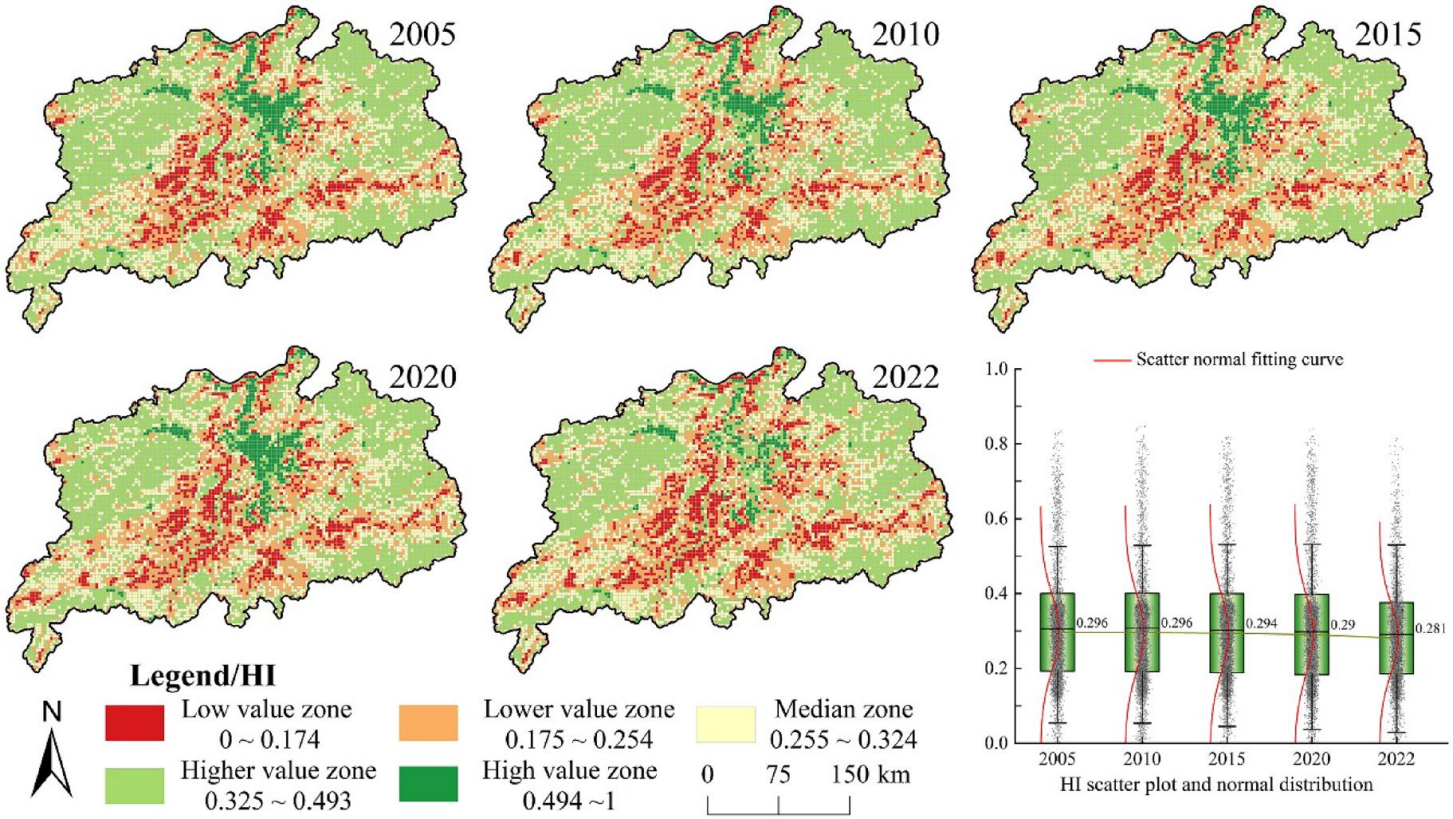
Ecosystem health level spatial differentiation in the Poyang Lake urban cluster. The process of creating Fig. 6 is as follows: First, calculate the ecosystem health level of the study area in ArcGIS 10.8 for the years 2005, 2010, 2015, 2020, and 2022, and export the results as five separate images. Then obtain the HI scatter plot and normal distribution in Origin 2023, and export the results as separate images. Finally, arrange and compile in Adobe Photoshop 2023 to generate Fig. 6.

With the above natural ecosystem health index and ecosystem service index, a comprehensive ecosystem health assessment was conducted in the region^23^. The assessment results were classified into five levels according to the actual conditions of the Poyang Lake urban agglomeration by referring to the established criteria for ecosystem health: low (0–0.174), relatively low (0.175–0.254), moderate (0.255–0.324), fairly good (0.325–0.493) and excellent (0.494–1.000) (Fig. 6). Spatially, the distribution of ecosystem health levels was similar to the ecosystem service index in the Poyang Lake urban agglomeration. The ecosystem health level was mostly ‘excellent’ across the region, whereas the surrounding areas, with this region at the centre, were large. Moreover, the areas at ‘low’ and ‘relatively low’ levels also tended to extend towards the southeast and southwest, highlighting an overall low health level and obvious spatial differentiation. Temporally, the mean ecosystem health index declined from 0.296 (2005) to 0.281 (2022), with intermediate values of 0.296 (2010), 0.294 (2015), and 0.290 (2020). While 78.18% of the area maintained stable health levels (primarily in the northeast and northwest), only 3.97% showed improvement (concentrated in the southeast). This assessment reveals the continuous high-intensity development in the urban agglomeration has reduced ‘excellent’ health areas, with expanding human impacts degrading ecosystem services across broader regions.

**Fig. 7:**
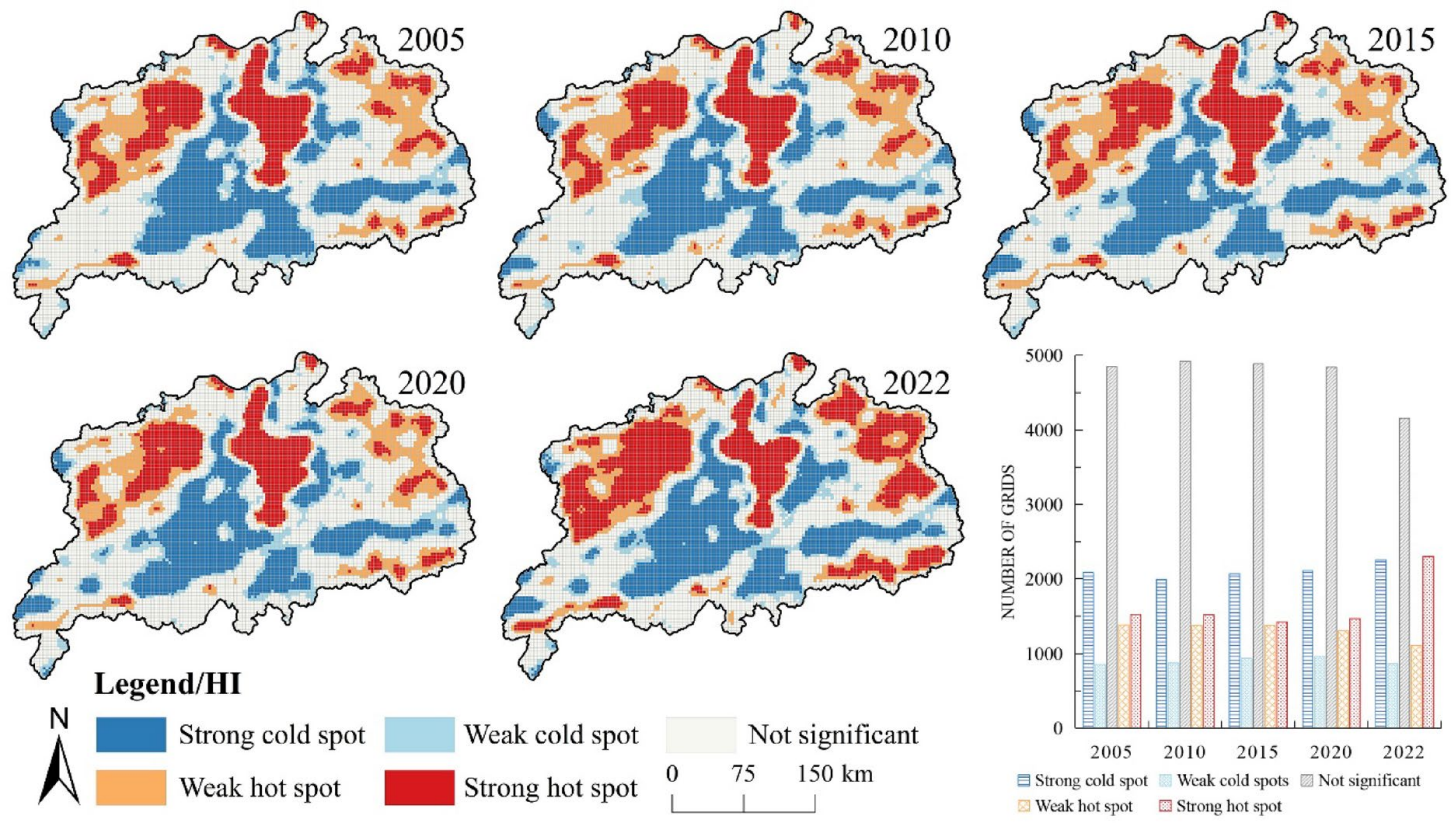
Cold and hot spot spatial distribution of ecosystem health level in the Poyang Lake urban cluster. The process of creating Fig. 7 is as follows: First, calculate the cold and hot spot spatial distribution of the ecosystem health level for the study area in ArcGIS 10.8 for the five years 2005, 2010, 2015, 2020, and 2022, and export the results as five separate images. Then obtain the bar chart of number of grids in Microsoft Office Excel 2016 and export the result as a separate image. Finally, arrange and compile in Adobe Photoshop 2023 to generate Fig. 7.

The results of the global Moran’s I for the ecosystem health level of the Poyang Lake urban agglomeration (Table 8) revealed a significant positive spatial agglomeration effect with values of 0.635, 0.612, 0.631, 0.642 and 0.569 for the years 2005, 2010, 2015, 2020 and 2022, respectively. However, the decreasing trend in Moran’s I suggested a diminishing neighbouring effect of ecosystem health. Meanwhile, the spatial hotspot analysis unveiled high–high and low–low adjacencies of the ecosystem health index (Fig. 7). Hot spots and minor hot spots were primarily concentrated in the Poyang Lake area, and the west of Jiujiang, with an expansion of hot spots also observed north of Jingdezhen, northeast of Shangrao and west of Jiujiang. Cold spots and minor cold spots were predominantly found east and southeast of Yichun and north of Fuzhou. There was a marked ‘polarisation’ in ecosystem health in the study area.

**Table 8:** Global autocorrelation test of ecosystem health status in the Poyang lake urban agglomeration.

HI	2005	2010	2015	2020	2022
Moran’s I	0.635	0.612	0.631	0.642	0.569
z scores	91.275	87.905	90.628	92.319	81.763
P value	<0.001	<0.001	<0.001	<0.001	<0.001

### Driving factors for the Spatial differentiation of ecosystem health in the Poyang lake urban agglomeration

Geodetector was chosen for its ability to quantify individual and interactive effects on spatial heterogeneity. Unlike traditional regression models, Geodetector explicitly evaluates the explanatory power of categorical variables through stratification, which aligns with the goal of identifying dominant drivers in a socio-natural coupled system characterized by nonlinear interactions. While the q-value indicates the explanatory power of a factor, it does not consider potential interactions between factors. To address this limitation, we incorporated interaction detection analysis to examine the combined effects of various factors.

#### Selection and analysis of driving factors

Referring to previous research findings^23^on the basis of the specific conditions of the Poyang Lake urban agglomeration, this study continued to explore the key driving factors for ecosystem health. With nine driving factors selected in total (Table 9), the driver behind the spatial differentiation and evolution of ecosystem health in the Poyang Lake urban agglomeration was analysed through factor detection and interactive factor detection. According to data availability, the ecosystem health level was designated as the dependent variable *Y*, whereas the annual precipitation (*X*1), annual average temperature (*X*2), net primary productivity of vegetation (*X*3), DEM (*X*4), slope (*X*5), average nighttime light index (*X*6), per capita farmland area (*X*7), population density (*X*8) and land urbanisation degree (*X*9) were selected as the independent variable *X*.

**Table 9:** Driving factors for the ecosystem health of the Poyang lake urban agglomeration.

Driving Factor	Factor	Unit	Number
Ecological function indicators	Annual average precipitation	mm	X1
	Annual average temperature	°C	X2
	Net primary productivity of vegetation	g/(m²·a)	X3
Ecological structure indicators	DEM	m	X4
	Slope	degrees per meter	X5
Socioeconomic indicators	Average nighttime light index	Lux	X6
	Per capita farmland area	hm^2^	X7
	Population density	people per square kilometer	X8
	Land urbanisation degree	%	X9

The analysis of driving factors revealed varying impacts on ecosystem health (Table 10). Across the study period (2005–2022), population density and land urbanisation degree consistently ranked as the top two drivers (q-values = 0.404 and 0.542, respectively, *p* < 0.01), demonstrating stronger explanatory power than ecological structure/function indicators. This dominance of socioeconomic indicators likely stems from urban expansion converting natural lands to built-up areas, which reduces ecosystem service capacity through habitat fragmentation and water pollution. Among ecological function indicators, annual average temperature exerted greater influence than net primary productivity of vegetation, while slope showed higher explanatory power than DEM among ecological structural indicators. Notably, the relative contribution of natural factors increased over time, coinciding with enhanced ecological conservation measures and diversified human activities that partially mitigated urbanization pressures.

**Table 10:** Results of driving factor detection for the Poyang lake urban agglomeration.

Driving Factor	Factor	q-value					
		2005	2010	2015	2020	2022	Five-year average
Ecological function indicators	Annual average precipitation	0.039***	0.030***	0.005***	0.029***	0.001*	0.021***
	Annual average temperature	0.082***	0.080***	0.077***	0.066***	0.100***	0.225***
	Net primary productivity of vegetation	0.035***	0.063***	0.055***	0.048***	0.023***	0.097***
Ecological structure indicators	DEM	0.077***	0.068***	0.073***	0.074***	0.100***	0.229***
	Slope	0.097***	0.095***	0.101***	0.105***	0.152***	0.251***
Socioeconomic indicators	Average nighttime light index	0.001	0.005***	0.016***	0.040***	0.000	0.399***
	Per capita farmland area	0.037***	0.031***	0.039***	0.025***	0.016***	0.133***
	Population density	0.282***	0.270***	0.286***	0.321***	0.343***	0.404***
	Land urbanisation degree	0.179***	0.176***	0.185***	0.199***	0.193***	0.542***

Note: *** *p* < 0.01, ** *p* < 0.05, * *p* < 0.1.

#### Interaction detection analysis

**Fig. 8:**
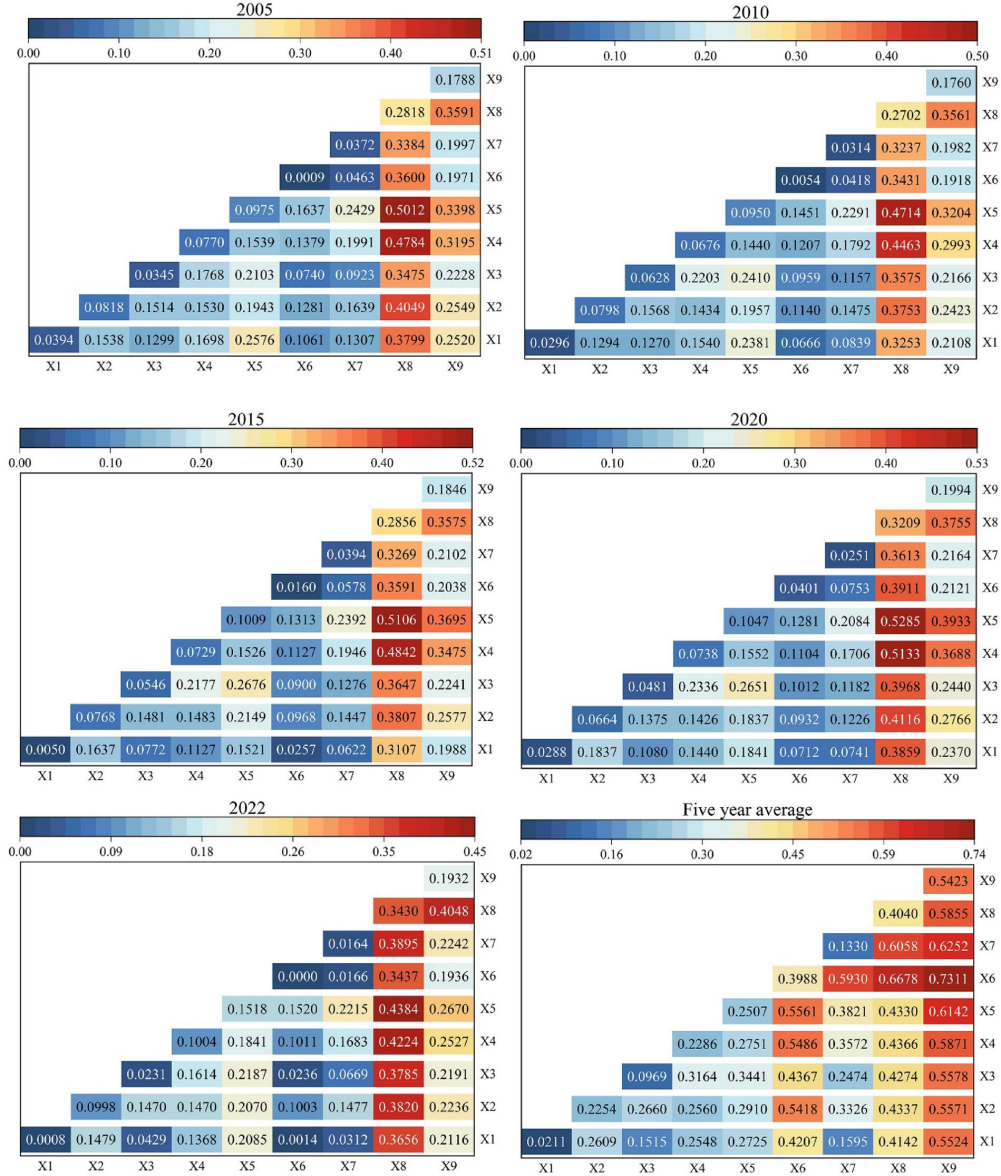
Results of the interaction detection of ecosystem health drivers in the Poyang Lake urban agglomeration.

Geodetector analysis revealed significant enhancement effects when considering factor interactions (Fig. 8). The combined q-values consistently exceeded individual factor impacts, demonstrating stronger explanatory power for ecosystem health variations. The most influential interactions in 2005 were slope ∩ population density (q = 0.5012), DEM ∩ population density (q = 0.4784), and annual temperature ∩ population density (q = 0.4049), maintaining strong explanatory power in different years, maintaining strong explanatory power in different years. Throughout the study period, interactions involving population density consistently showed the highest explanatory power, followed by those involving the degree of land urbanisation. The *q*-value increased significantly when these two factors interacted with each other. Among all the outcomes of interaction detection, the average nighttime light index ∩ degree of land urbanisation ranked top in terms of average explanatory power (0.7311), followed by the average nighttime light index ∩ population density (0.6678) and slope ∩ land urbanisation degree (0.6142). The nighttime light index is a proxy for urban development and economic activity, reflecting the intensity of human-induced disturbances. Therefore, the interactions between socioeconomic indicators (e.g. population density and land urbanisation) and ecological indicators (e.g. annual average temperature, DEM and slope) exerted varying degrees of influence on the ecosystem health of the Poyang Lake urban agglomeration, exacerbated spatiotemporal disparities and altered regional ecosystem structures and functions. With varied driving effects on ecosystem health in the Poyang Lake urban agglomeration, such interactions influenced the security and stability of the regional ecosystem. Future urban planning policies can integrate ecosystem health assessments to ensure sustainable development and ecological balance.

### Types of level changes and zoning control of ecosystem health

**Fig. 9:**
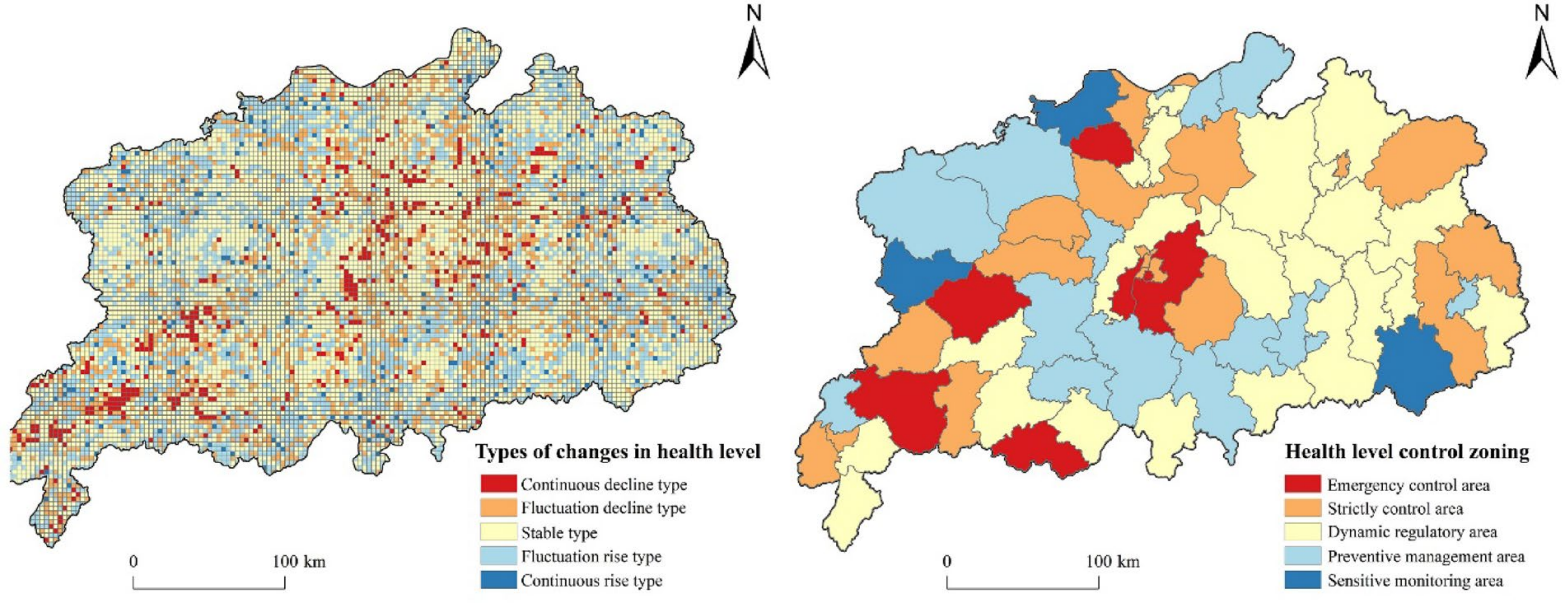
Types and zoning control of ecosystem health level in the Poyang Lake urban agglomeration. The process of creating Fig. 9 is as follows: First, Types of changes in health levels are calculated in ArcGIS 10.8 for each grid in the study area, and the results are exported as separate images. Then health level zoning control is calculated in ArcGIS 10.8 on a county by county basis to determine its Control Zones, and the results are exported as separate images. Finally, arrange and compile in Adobe Photoshop 2023 to generate Fig. 9.

The Poyang Lake urban agglomeration was divided into five types of areas according to the characteristics of the changes in the comprehensive ecosystem health levels (Fig. 9). The results revealed that the Poyang Lake urban agglomeration exhibited distinct spatial patterns in comprehensive ecosystem health changes, with stable areas dominating (49.61% coverage) while showing notable regional variations. Areas experiencing continuous decline (4.62%) concentrated in Nanchang’s urban core, central Yuanzhou District, and southern Yifeng County, whereas fluctuating declines (16.05%) appeared around Poyang Lake. Northwestern and northeastern regions including Wuning, Ruichang and Wuyuan Counties demonstrated fluctuating improvement (27.55%), while minimal continuous improvement areas (2.17%) showed no discernible spatial agglomeration.

To effectively manage and control the health level of the urban agglomeration, five types of control zones were tailored for the current ecosystem health and types of changes in the Poyang Lake urban agglomeration, with counties as a unit, based on the urban comprehensive health levels and changing trends. The emergency control zone encompassed the Honggutan District, Nanchang County and Yuanzhou District, accounting for 15.63%. This zone, at a low and declining health level, requires immediate ecological protection and governance. The strict control zone was primarily situated in central areas under rapid urbanisation, with a percentage of 26.56%, requiring steady progress in ecological security management and control to ensure sustainable urbanisation and ecosystem health. The dynamic regulation zone, accounting for the largest proportion of ~ 28.13%, was mainly located northeast and southeast of the urban agglomeration. The preventive management zone was primarily distributed south and northwest of the urban agglomeration, accounting for ~ 26.56%, playing a vital role in conserving soil and water and maintaining biodiversity. The sensitive monitoring zone focused on areas with high current health levels but significant changes in the level type, which, with a percentage of only 3.12%, were distributed in Ruichang, Qianshan County, Tonggu County and other places.

The key to accelerating modernisation with mankind and nature in harmonious coexistence is balancing development and ecology^53^. A holistic and coordinated approach should be adopted to synergistically promote high-quality economic development and high-standard ecological protection. On the basis of the above classification of control zones, differentiated strategies are presented: (1) In the emergency control zone under rapid urbanisation, attention should also be given to other control areas besides lake reclamation to promote green transformation, strengthen ecological protection and maintain watershed connectivity. (2) In the strict control zone with frequent human activities and high-intensity land development, natural ecological security boundaries should be guarded, territorial space should be optimised, and low-carbon development should be promoted. (3) The expansive dynamic regulation zone requires identifying areas with significant conflicts between development and protection, strengthening resource conservation, regulating development and ensuring ecological quality. (4) The preventive management zone, hosting critical ecological spaces, should have enhanced prevention and early warning mechanisms, play a fundamental role in the source prevention system for ecology and environment and provide strong support for sustainable development. (5) The sensitive monitoring zone should prioritise ecological protection and restoration, allowing for development while ensuring risk control.

## Discussion and conclusion

### Discussion

From a spatiotemporal dynamic perspective, this paper studied the Poyang Lake urban agglomeration and built a VORS ecosystem health assessment framework to assess and analyse the spatiotemporal variation characteristics of the region over the years 2005, 2010, 2015, 2020 and 2022. Moreover, Geodetector was utilised to identify key driving factors influencing ecosystem health in this region and conduct an in-depth analysis of their interactions. A level–type approach was adopted for zoning and formulation of strategies to provide a reference and suggestions for ecosystem management of the urban agglomeration. In terms of limitations, there is room for innovation in quantifying the relationship between ecosystem services and ecosystem health, as well as a lack of consideration for the ecological health perspective under the influence of natural attributes using the equivalent factor method. In the future, the breadth and depth of ecosystem health assessment will be further expanded.

#### Evolution analysis of ecosystem health in the Poyang lake urban agglomeration

The spatial-temporal analysis of the Poyang Lake urban agglomeration (2005–2022) observed “high-mountainous flanks, low-central plains” gradient in ecosystem health aligns with the VORS framework’s^57^ emphasis on the interplay between vigor (NDVI stability in mountainous areas), organization (connectivity loss in fragmented plains), and resilience (45.06–46.69% areas maintaining stability). This pattern further reflects the ecosystem service cascade model, where land-use pressures disrupt the “natural capital to service flow” chain, particularly in economically vibrant zones(11.10%degradation).While mountainous regions retained relatively stable ecological conditions, the central plains exhibited intensified fragmentation^58^,likely driven by asymmetric land use transitions​​:urban expansion and agricultural intensification reduced natural habitats, aligning with trends observed in other rapidly urbanizing basins^59^.Notably, ecosystem service capacity displayed spatial heterogeneity, with 11.10%of areas experiencing degradation—predominantly in economically vibrant zones—versus 3.03%improvement in peripheral regions^60^.This divergence underscores the trade-offs between socioeconomic growth and ecological resilience, where heightened anthropogenic pressures may exceed localized carrying capacities^61^.However, the comprehensive ecosystem health index demonstrated moderate stability, with 45.06–46.69% of areas consistently exceeding annual averages, suggesting partial success of existing conservation measures.These findings highlight the need to integrate VORS-based assessments with cascade-driven management for balanced socio-ecological outcomes.

#### Typical driving factors for ecosystem health in the Poyang lake urban agglomeration

The geodetector analysis revealed that population density and land urbanization rate were the most influential socioeconomic factors affecting ecosystem health in the Poyang Lake Urban Agglomeration. Increased population density intensifies human activities, potentially leading to overexploitation of natural resources, environmental degradation, and subsequent ecological pressures such as pollution escalation, habitat fragmentation, and biodiversity loss^62^. Interaction factor detection further identified annual average temperature, DEM, slope gradient, and population density as primary drivers through their synergistic effects. These interactions likely shape the composition, functionality, and spatial evolution of regional ecosystems during urbanization, a finding consistent with previous studies^63,64^.

#### Types of changes and zoning control of ecosystem health in the Poyang lake urban agglomeration

The dynamic changes in ecosystem health across the Poyang Lake urban agglomeration were categorized into five distinct classes, revealing spatial resilience and localized vulnerabilities. While the overall health level remained relatively stable, urban cores exhibited increased sensitivity to ecological pressures, necessitating targeted interventions in rapidly urbanizing zones^6^. Approximately 14% of areas showed continuous decline, predominantly in high-development corridors, whereas fluctuating zones highlighted the dynamic interplay of natural variability and anthropogenic influences. Notably, 68% of the region maintained stable ecosystem health, suggesting that current conservation measures partially mitigate urbanization impacts.

To address these patterns, a five-tier zoning framework (Emergency Control, Strict Control, Dynamic Regulation, Preventive Management, Sensitive Monitoring) was developed and tailored​​ to regional priorities. The five-tier zoning framework’s county-level implementation aligns with China’s administrative hierarchy, where counties serve as the smallest complete governance unit with independent legislative and fiscal capacities. This approach balances ecological integrity with administrative feasibility. The zoning integrates “Ecological Function Zoning” principles by prioritizing ecosystem services while accommodating “Major Function-Oriented Planning” through differentiated development intensities. For instance, Emergency Control Zones can balance growth and biodiversity, drawing from the successful implementation of the Ecological Conservation Redline (ECR) policy in the Yangtze River Delta^65^. Dynamic Regulation Zones can employ wetland buffers to mitigate habitat encroachment, consistent with Jiangxi’s ‘no net loss’ wetland mandate under the Jiangxi Provincial Wetland Protection Regulations (2021)^66^. These strategies integrate national guidelines (e.g., National Ecological Function Zoning) with provincial regulations, reflecting stakeholder consensus on adaptive governance^67^. Future work will integrate stakeholder interviews with nested governance approaches, combining county-led enforcement and basin-wide coordination committees to enhance zoning practicality while addressing policy fragmentation.

### Conclusions

(1) The natural ecosystem health in the Poyang Lake urban agglomeration showed high values in mountainous regions and low values in the central plain. The mean natural ecosystem health index declined during the study period, with 14.70% of grid cells decreasing from 2005 to 2022, mainly in central Nanchang, southeast Yichun and north Yingtan. The ecosystem service index had high values around Poyang Lake and low values in surrounding areas, with 41.48% of grid cells declining. Only 14.13% of grid cells experienced changes in the ecosystem service index level, including 11.10% decline and 3.03% increase.

(2) Ecosystem health in the Poyang Lake urban agglomeration was generally low and spatially differentiated, with a notable polarization. Poyang Lake had excellent ecosystem health, while surrounding areas mostly had low or very low levels. 78.18% of grid cells had unchanged health levels, 3.97% improved and 17.85% declined. Hot spots were mainly in the Poyang Lake area and west of Jiujiang, expanding over time. Cold spots were mainly southeast of Yichun and north of Fuzhou.

(3) The average nighttime light index most significantly influenced the evolution of ecosystem health levels in the Poyang Lake urban agglomeration. Interactive factor detection results in 2005, 2010, 2015, 2020 and 2022 showed that interactions between annual mean temperature, DEM, slope, population density and other factors were the main drivers affecting ecosystem health, influencing changes during urbanization.

(4) Ecosystem health changes in the study area were generally stable but varied significantly in some parts. Areas with continuous decline were mainly in Nanchang urban area, central Yuanzhou District and south of Yifeng County, accounting for 4.62% of the total area. Stable areas, constituting 49.61%, indicated effective ecological management. Areas with fluctuating decline and increase, accounting for 16.05% and 27.55% respectively, highlighted the need for dynamic monitoring and timely intervention. Continuously increasing areas, mainly farmlands and forestlands, were scattered and accounted for 2.17%.

## Electronic supplementary material

Below is the link to the electronic supplementary material.

Supplementary Material 1

## Data Availability

The datasets generated and/or analysed during the current study are not publicly available due part of the data comes from government agencies ，but are available from the corresponding author on reasonable request.
