# Supplementary material for: Dynamic assessment and zoning strategies for ecosystem health in Poyang lake urban agglomeration
Source: Sci Rep. 2025 Jul 1;15:21956. doi: 10.1038/s41598-025-05776-6 (PMC12219720; doi:10.1038/s41598-025-05776-6)
Supplement: Supplementary file 1 — Supplementary Material 1 [file 41598_2025_5776_MOESM1_ESM.docx]

**Supplementary Materials**

# Submission ID:b4ee3529-b335-4b38-a673-f66bb88c3e10

**Title:Dynamic Assessment and Zoning Strategies for Ecosystem Health in Poyang Lake Urban Agglomeration**

**Table 1.** Data source and precision

| Type | Formats | Precision | Data source | References |
| --- | --- | --- | --- | --- |
| Land use/land cover data | Raster | 30m | <https://zenodo.org/record/8176941> | [1] |
| Normalized Difference Vegetation Index(NDVI) | Raster | 1km | <https://e4ft101.cr.usgs.gov/> | [2] |
| Net primary productivity of vegetation(NPP) | Raster | 500m |  | [3] |
| Annual average precipitation | Raster | 1km | <https://data.tpdc.ac.cn/> | [4] |
| Annual average temperature | Raster | 1km |  |  |
| Average nighttime light index | Raster | 1km | http://www.resdc.cn/ | \ |
| Digital elevation model | Raster | 30m | https://panda.copernicus.eu/panda | \ |
| Population density | Raster | 1km | https://landscan.ornl.gov/ | \ |
| Administrative district | Vector | \ | https://www.webmap.cn/main.do?method=index | \ |
| Other socio-economic data | Panel data | \ | Corresponding city, county, and district statistical yearbooks and  consultation with local governments | \ |

**References:**

[1]Yang, J., & Huang, X. (2021). 30 m annual land cover and its dynamics in China from 1990 to 2019. doi:10.5194/essd-2021-7.

[2]Running, S., Mu, Q., & Zhao, M. (2021). MODIS/Terra Gross primary productivity 8-day L4 Global 500m SIN Grid V061 . NASA EOSDIS land processes distributed active archive center. doi:10.5067/MODIS/MOD17A2H.061

[3]Didan, K. (2015). MOD13A3 MODIS/Terra Vegetation indices monthly L3 global 1km SIN grid V006. NASA EOSDIS Land Processes Distributed Active Archive Center. doi:10.5067/MODIS/MOD13A3.006

[4]Shouzhang Peng. (2019). High-spatial-resolution monthly temperatures dataset over China during 1901–2017. doi:10.5281/ZENODO.3185722.

**Table 2. Analytical methods for driving factors and spatial heterogeneity**

| **Specific Research Methods** | **Equation** | **Definition** | **Geographical Significance** | **Role** | **References** |
| --- | --- | --- | --- | --- | --- |
| Global Moran’s I | $I=\frac{n\sum_{i=1}^{n} \sum_{j=1}^{n} W_{ij}(x_{i}-\overline{x})(x_{j}-\overline{x})}{\sum_{i=1}^{n} \sum_{j=1}^{n} {W_{ij}(x_{i}-\overline{x})}^{2}}$ | *x_i_* and *x_j_* represent the ecosystem health values in regions *i* and *j*, respectively; *x*ˉ denotes the mean value; *W_ij_* is the spatial weight matrix; *n* represents the number of grid cells. | When *I* > 0, there is a positive correlation among ecosystem health levels; when *I* < 0, there is a negative correlation among ecosystem health levels; and when *I* = 0, the distribution is independent and random. | Global Moran’s I is a statistical measure used to assess the degree of spatial autocorrelation across an entire study area. It determines whether observed spatial patterns (e.g., clustering or dispersion) deviate significantly from randomness. | [1,2] |
| Hotspot analysis | $G_{i}^{*}=\frac{\sum_{j=1}^{n} W_{ij}-\overline{x}\sum_{j=1}^{n} W_{ij}}{S\sqrt{\frac{\left[ n\sum_{j-1}^{n} W_{ij}^{2}-\left( \sum_{j=1}^{n} W_{ij} \right)^{2} \right]}{n-1}}}$ | 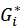 denotes the local autocorrelation index for region *i*, where *n* is the number of assessment units, *x_j_* represents the index value for unit *j*, *W_ij_* is the spatial weight matrix, *S* is the standard deviation, and 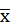 is the mean value. | 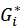 > 0 indicates that the region is an aggregation of hot spots; 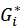 < 0 suggests that the region is a negative aggregation of hot spots; 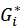 = 0 implies that the result is randomly generated and statistically insignificant. | This method identifies localized spatial clusters (hotspots or coldspots) where values are statistically higher or lower than expected under spatial randomness. | [3,4] |
| Geodetector | $q=1-\frac{1}{N\sigma^{2}}\sum_{h=1}^{L} N_{h}{\sigma_{h}}^{2}$ | *q* represents the influence of a certain factor on the spatial differentiation of ecosystem health; *σ*²*h* denotes the variance of the assessment units, with *σ*² as the variance across the entire region; *N* is the number of assessment units in the study area. | The value range of *q* is [0,1], with a higher value indicating a greater influence on the spatial differentiation of ecosystem health. | Geodetector evaluates the explanatory power of driving factors and their interactions on spatial phenomena, leveraging spatial stratified heterogeneity principles. | [5,6,7] |

**References:**

[1]Chen, W., Wang, G. & Zeng, J. Impact of Urbanization On Ecosystem Health in Chinese Urban Agglomerations. Environ. Impact Assess. Rev. 98, 106964 (2023).

[2] Luo, S., Luo, Z., Yang, X., Zhang, F. & Lei, Y. Changes in Ecosystem Services and Equity Identification in Nanchang Under the Influence of Construction Land Expansion. Environmental Science. 1-22 (2024).

[3] Luo, S. & Luo, Z. Spatial Differentiation and Associated Factors of Non-Grain Cultivated Land in Mineral Grain Composite Area Considering Scale Effects. Transactions of the Chinese Society of Agricultural Engineering. 40, 265-275 (2024).

[4]Ouyang, N., Rui, X., Zhang, X., Tang, H. & Xie, Y. Spatiotemporal Evolution of Ecosystem Health and its Driving Factors in the Southwestern Karst Regions of China. Ecol. Indic. 166, 112530 (2024).

[5]Qiu, Z. et al. Spatiotemporal Analysis of the Interactions Between Ecosystem Services in Arid Areas and their Responses to Urbanization and Various Driving Factors. Remote Sensing, 2024.

[6]Wang, J. & Xu, C. Geodetector: Principle and Prospective. Acta Geographica Sinica. 72, 116-134 (2017).

[7]Shen, W., Zheng, Z., Qin, Y. & Li, Y. Spatiotemporal Characteristics and Driving Force of Ecosystem Health in an Important Ecological Function Region in China. Int. J. Environ. Res. Public Health. 17, (2020).
